# Sex differences in risk factors for stroke in patients with hypertension and hyperhomocysteinemia

**DOI:** 10.1038/s41598-019-50856-z

**Published:** 2019-10-04

**Authors:** Hui Pang, Qiang Fu, Qiumei Cao, Lin Hao, Zhenkun Zong

**Affiliations:** 1 Department of Cardiology, Xuzhou Central Hospital, Xuzhou Clinical School of Xuzhou Medical University, Xuzhou, Jiangsu China; 2 Department of Urinary Surgery, Xuzhou Central Hospital, Xuzhou, Jiangsu China; 3 Department of Neurosurgery, Affiliated Hospital of Xuzhou Medical University, Xuzhou, Jiangsu China

**Keywords:** Cardiovascular genetics, Stroke

## Abstract

Data on the sex-specific differences in risk of stroke among patients with H-type hypertension are limited. We aimed to analyze interactions between sex and other risk factors on stroke, including the sex-methylenetetrahydrofolate reductase (MTHFR) interaction. A retrospective analysis of baseline data from 2040 patients with hypertension and hyperhomocysteinemia (HHcy) included demographic characteristics, biomarkers, history of chronic diseases and lifestyle factors. Polymerase chain reaction-restriction fragment length polymorphism method was used to investigate the C677T polymorphism of MTHFR gene. We examined independent effects and interactions between sex and stratified factors on the risk of stroke by logistic regression model. A total of 1412 patients suffered stroke, and the prevalence of stroke was 70.65% in men and 66.53% in women. Both men and women had independent risk factors for stroke, including diabetes mellitus, atrial fibrillation, smoking, increased level of systolic blood pressure (SBP) and plasma total homocysteine (tHcy), as well as the decreased level of high-density lipoprotein cholesterol. Diastolic blood pressure (DBP) -specific risk of stroke was unique to men. Interactions between sex and other risk factors on stroke risk were statistically significant: age, fasting plasma glucose (FPG), SBP, DBP, triglycerides (TG) and tHcy. Furthermore, tHcy interacted with age, SBP and DBP in men, and age, SBP, DBP, FPG, and TG in women to modulate the risk of stroke. Although TT genotype did not have an independent effect on stroke, it could interact with sex and FPG, TG and SBP to increase stroke. In conclusion, sex-specific differences are useful to stratify the risk of stroke and assist clinicians in the decision to select a reasonable therapeutic option for high-risk patients.

## Introduction

Stroke, a type of cerebral impairment syndrome induced by acute blood circulation in brain, is one of the leading causes of mortality and morbidity, with Asian disproportionately affected^1^. To date, the global burden of management for patients with stroke is mainly coming from Asia due to rise in the prevalence of vascular risks. In China, stroke featured by high prevalence, mortality, morbidity and recurrence, is considered as the leading cause for death and disability^2^. In general, the male population are prone to develop stroke compared to the female counterparts, however, a higher incidence of disability is reported in women and the possibility of functional independence is comparatively lower^3^. Sex differences in stroke risk and prognosis may be associated with the genetic backgrounds, including immune system^4^, coagulation^5^, endocrine system^6^, reproductive system, as well as social factors.

Hyperhomocysteinemia (HHcy) defined as the concentration of plasma total homocysteine (tHcy) of ≥10 μmol/L^7^, is often a consequence of reduced enzymatic activity involved in homocysteine metabolism, such as a point mutation of methylenetetrahydrofolate reductase (MTHFR) in which cytosine is replaced by thymidine at position 677 (MTHFR C677T)^8^. H-type hypertension, which refers to concurrence of primary hypertension and HHcy, is an important factor for increased prevalence and persistent progression of stroke in China mainland^7^. Previous studies have shown that hyperhomocysteinemia (HHcy) and hypertension often coexist. HHcy and hypertension are two independent, modifiable risk factors for incident stroke and stroke death. Moreover, coexistence of HHcy and hypertension could act additively on a multiplicative scale, which contributed to the risk of stroke and stroke-related death^9^.

Many studies have shown sex differences in the association between Hcy and different diseases. A study from the Korea that included 150 men and 132 women showed that Hcy was associated with the prevalence of non-alcoholic fatty liver disease in men but not in women^10^. Chen *et al*. conducted a cross-sectional study, and observed that Hcy was negatively associated with estimated glomerular filtration rate, which was more profound in women^11^. Sex differences in the association between traditional risk factors besides MTHFR C677T genotype and stroke in patients with H-type hypertension remain unsettled and include the possibility of a sex interaction. The objective of this study was to investigate sex differences in independent effect of risk factors on stroke and important interactions between individual factors by subgroup analysis.

## Subjects and Methods

### Study design

This was a retrospective study among 2040 hospitalized patients with H-type hypertension (men: 1329; women: 711; age: 61.02 ± 8.68 years) recruited continuously from October 2013 to October 2014 in Brain Hospital, Affiliated Hospital of Xuzhou Medical University and department of Cardiology, Xuzhou Central Hospital. Individuals with a systolic blood pressure (SBP) of 140 mm Hg or higher or a diastolic blood pressure (DBP) of 90 mm Hg or higher at rest, or with definite medical records for hypertension and current administration with antihypertensive drugs, were all diagnosed as hypertension. It includes graded 1 (140–159 mmHg SBP and/or 90–99 mmHg DBP), 2 (160–179 mmHg SBP and/or 100–109 mmHg DBP), and 3 (≥180 mmHg SBP and/or ≥110 mmHg DBP)^12^. Types of stroke included cerebral ischemic stroke, hemorrhagic stroke and transient ischemic attack (TIA). Stroke is based on strict neurological examination and computer tomography scans or magnetic resonance imaging^13^. Confirmation of dyslipidemia is in accordance with the 2017 American Association of Clinical Endocrinologists and American College of Endocrinology guidelines for the management of dyslipidemia and prevention of cardiovascular disease. Triglyceride (TG) levels <1.7 mmol/L are normal, 1.7 to 2.3 mmol/L are borderline-high and ≥2.3 mmol/L are high. Total cholesterol (TC) levels <5.2 mmol/L are normal, ≥5.2 and <6.2 mmol/L are borderline-high and ≥6.2 mmol/L are high^14^. Diabetes mellitus (DM) is diagnosed based on the fasting plasma glucose (FPG) ≥7.0 mmol/L. When the FPG concentration is ≥6.1 and <7.0 mmol/L, impaired fasting glucose (IFG) should be considered^15^. Body mass index (BMI) level <24.0 kg/m^2^ is considered “normal” for adults, with 24.0 to 27.9 kg/m^2^ considered “overweight” and 28.0 kg/m^2^ or greater considered “obesity”^16^. The cutoff values for age are based on recommendations from the Global Burden of Disease Study 2010^17^. Those with secondary hypertension, acute or chronic inflammation, primary nephritis, arthritis, and systemic lupus erythematosus were excluded from the study. Study protocols were approved by the Ethics Committee of Xuzhou Central Hospital, and each patient signed the informed consent. All methods were performed in accordance with relevant guidelines and regulations.

### Data collection and blood chemical assay

The collected clinical data included age, sex, BMI, cigarette smoking, alcohol consumption, SBP, DBP, as well as history of chronic diseases, such as stroke, coronary artery disease (CAD), DM, hyperlipidemia, and atrial fibrillation (AF). Fasting venous blood was collected from each participant within 24 hours after admission to measure FPG, TG, TC, high-density lipoprotein cholesterol (HDL-C) and tHcy. tHcy was determined using fluorescence polarization immunoassay method. Commercial kits used for the determination were purchased from the Abbott Corporation (CA, US).

### C677T Polymorphism of the MTHFR Gene

Genomic DNA was extracted from peripheral venous blood anticoagulated by EDTA using a GenElute^™^ Blood Genomic DNA Kit (Sigma-Aldrich Chemical Corporation, St. Louis, MO, US), according to manufacturer’s protocol. Genotyping C677T polymorphism of MTHFR gene was investigated using polymerase chain reaction-restriction fragment length polymorphism (PCR-RFLP) method. A 198 bp fragment was amplified by PCR using oligonucleotide primer sequences forward 5′- TGAAGGAGAAGGTGTCTGCGGGA-3′ and reverse 5′-AGGACGGTGCGGTGA GAGTG-3′. Amplified PCR products were digested with HinfI restriction enzyme (Promega Corporation, Madison, US). Mutation at position 677, where C was replaced by T, created a restriction site for the enzyme. The wild-type (CC) genotype produced a 198 bp fragment. Heterozygous (CT) genotype produced two fragments: 198 bp and 175 bp, and the homozygous mutant (TT) produced a 175 bp fragment. For verifying the genotyping results of PCR-RFLP, 600 subjects with different genotypes were randomly selected for DNA sequencing analysis, with 100% agreement.

### Statistical analysis

Analyses were performed by using SPSS 20.0 software. Continuous variables were presented as mean ± standard deviation. Categorical variables were presented as number of cases (n) and percentage. tHcy was log transformed to reduce skewness and heteroscedasticity. Sex differences in demographic characteristics of the participants were compared using Chi-square tests for categorical factors and Student’s t-tests for continuous variables. Using Kruskal-Wallis test, we can assess intergroup significance among the stratified factors. Multiple binary logistic regression models were constructed using the forced entry method to evaluate the associations between stroke risk and risk factors, interactions between tHcy and other risk factors on stroke risk by sex in patients with H-type hypertension. The models had adjustment for age, BMI, SBP, DBP, FPG, TG, TC, HDL-C, tHcy, smoking, alcohol consumption, MTHFR C677T polymorphism, and comorbidity. We defined comorbidity as a history of a diagnosis of 1 of the following previously reported concomitant risk factors: DM, hyperlipidemia, CAD, or AF. Binary variables for each of these factors and MTHFR C677T polymorphism were included as dummy variables in the models. Interactions between sex and age, BMI, FPG, SBP, DBP, TG, TC, tHcy and MTHFR C677T polymorphism on stroke risk were also individually tested by adding multiplicative term of sex and stratified factors besides all baseline variables to multiple binary logistic regression models. Interaction between each subgroup factor and sex was tested for differences in stroke risk among a subset of individuals with known CT/TT subtypes compared with CC subtype. Factors of subgroup analyses included age (≥55 years *vs*. <55 years), BMI (≥28 kg/m^2^ *vs*. <28 kg/m^2^), FPG (≥6.1 mmol/L *vs*. <6.1 mmol/L), SBP (≥160 mmHg *vs*. <160 mmHg), DBP (≥90 mmHg *vs*. <90 mmHg), TG (≥2.3 mmol/L *vs*. <2.3 mmol/L), TC (≥5.2 mmol/L *vs*. <5.2 mmol/L), and tHcy [≥18.0/(13.1–17.9) μmol/L *vs*. ≤13.0 μmol/L]. In these models, we additionally adjusted for sex. The results were expressed as coefficient, standard error (SE), odds ratio (OR) and 95% confidence interval (CI). All tests were 2-sided and *P* < 0.05 was considered statistically significant.

## Results

### Sex differences in patient demographics

Among the 2040 patients with H-type hypertension, FPG, TG, TC, and HDL-C levels in women were significantly higher than those of men, and the prevalence of hyperlipidemia was 52.32% and 45.82% for women and men, respectively. However, plasma tHcy (logarithmic value, Lg tHcy) level in women was significantly lower than that in men, as was also their exposure to environmental cigarette smoking and alcohol consumption. No statistical sex differences were observed in the age, SBP, DBP, BMI, genotype distributions of the MTHFR C677T polymorphism, and prevalence of stroke, CAD, DM and AF (*P* > 0.05, Table 1).

**Table 1:** Sex differences in demographic characteristics of patients with H-type hypertension.

Variables	Total	Men	Women	*P*-value
	(n = 2040)	(n = 1329)	(n = 711)	
Age, years	61.02 ± 8.68	60.95 ± 8.91	61.14 ± 8.22	0.642
BMI, kg/m^2^	24.72 ± 3.44	24.66 ± 3.29	24.82 ± 3.70	0.315
SBP, mmHg	156.17 ± 23.97	155.59 ± 23.54	157.27 ± 24.72	0.130
DBP, mmHg	92.36 ± 13.64	92.74 ± 13.68	91.65 ± 13.56	0.083
FPG, mmol/L	6.35 ± 2.10	6.26 ± 1.92	6.52 ± 2.39	0.010
TG, median (interquartile range)	1.63 (1.17–2.31)	1.57 (1.14–2.24)	1.75 (1.23–2.43)	0.001
TC, mmol/L	4.88 ± 1.01	4.72 ± 0.94	5.19 ± 1.07	<0.001
HDL-C, mmol/L	0.95 ± 0.30	0.91 ± 0.28	1.01 ± 0.32	<0.001
Lg tHcy	1.23 ± 0.20	1.26 ± 0.21	1.18 ± 0.15	<0.001
History of, no. (%)				
Cigarette smoking	542 (26.57)	492 (37.02)	50 (7.03)	<0.001
Alcohol consumption	476 (23.33)	449 (33.78)	27 (3.80)	<0.001
DM	539 (26.42)	334 (25.13)	205 (28.83)	0.071
Hyperlipidemia	981 (48.09)	609 (45.82)	372 (52.32)	0.005
CAD	379 (18.58)	240 (18.06)	139 (19.55)	0.409
AF	49 (2.40)	31 (2.33)	18 (2.53)	0.780
Stroke	1412 (69.22)	939 (70.65)	473 (66.53)	0.054
MTHFR C677T Polymorphism				0.054
CC	582 (28.53)	374 (28.14)	208 (29.26)	
CT	966 (47.35)	653 (49.14)	313 (44.02)	
TT	492 (24.12)	302 (22.72)	190 (26.72)	

Abbreviations: BMI, body mass index; SBP, systolic blood pressure; DBP, diastolic blood pressure; FPG, fasting plasma glucose; TG, triglycerides; TC, total cholesterol; HDL-C, high-density lipoprotein cholesterol; Lg tHcy, Log(10) total homocysteine; DM, diabetes mellitus; CAD, coronary artery disease; AF, atrial fibrillation.

### Sex differences in stroke risk factors

Binary Logistic regression analysis was carried out to assess the associations between stroke risk and risk factors by sex in patients with H-type hypertension, after adjustment for MTHFR C677T polymorphism and other conventional risk factors. Independent risk factors for stroke in both men and women included DM, AF, smoking, increased level of SBP and tHcy, as well as the decreased level of HDL-C. However, DBP elevation was an independent risk factor for stroke in men, but not in women (Table 2).

**Table 2:** Associations between stroke risk and risk factors by sex in patients with H-type hypertension.

Variables	Men, n = 1329				Women, n = 711			
	Coefficient	SE	OR (95%CI)	*P*-value	Coefficient	SE	OR (95%CI)	*P*-value
Age	−0.014	0.009	0.986 (0.969–1.003)	0.108	0.012	0.013	1.012 (0.987–1.038)	0.346
BMI	0.024	0.042	1.024 (0.944–1.112)	0.563	0.016	0.073	1.016 (0.881–1.172)	0.823
SBP	0.045	0.005	1.047 (1.036–1.057)	<0.001	0.035	0.006	1.036 (1.024–1.048)	<0.001
DBP	0.025	0.008	1.025 (1.009–1.042)	0.002	0.020	0.011	1.020 (0.999–1.041)	0.068
FPG	−0.026	0.053	0.975 (0.879–1.080)	0.623	0.101	0.072	1.107 (0.962–1.274)	0.157
TG	−0.071	0.092	0.932 (0.778–1.117)	0.445	0.130	0.125	1.139 (0.892–1.454)	0.297
TC	0.163	0.139	1.177 (0.896–1.545)	0.242	0.037	0.172	1.037 (0.741–1.453)	0.831
HDL-C	−1.708	0.288	0.181 (0.103–0.318)	<0.001	−1.479	0.343	0.228 (0.116–0.446)	<0.001
tHcy	0.019	0.006	1.019 (1.007–1.031)	0.001	0.049	0.014	1.050 (1.022–1.080)	<0.001
Cigarette smoking	0.949	0.143	2.583 (1.952–3.417)	<0.001	1.119	0.484	3.061 (1.185–7.907)	0.021
Alcohol consumption	0.238	0.178	1.269 (0.896–1.796)	0.180	−0.400	0.516	0.670 (0.244–1.842)	0.438
DM	0.847	0.239	2.333 (1.461–3.726)	<0.001	0.925	0.319	2.522 (1.351–4.709)	0.004
Hyperlipidemia	−0.238	0.178	0.788 (0.557–1.116)	0.180	0.038	0.242	1.039 (0.646–1.670)	0.876
CAD	0.052	0.193	1.053 (0.722–1.537)	0.788	0.125	0.250	1.134 (0.694–1.852)	0.616
AF	1.741	0.677	5.702 (1.513–21.494)	0.010	2.193	1.108	8.964 (1.022–78.603)	0.048
**MTHFR C677T Polymorphism**								
TT (*vs*. CC)	0.019	0.214	1.019 (0.670–1.550)	0.930	−0.067	0.263	0.935 (0.558–1.565)	0.798
CT (*vs*. CC)	0.042	0.167	1.042 (0.752–1.445)	0.803	0.283	0.229	1.327 (0.847–2.079)	0.218

Abbreviations: SE, standard error; OR, odds ratio; CI, confidence interval; BMI, body mass index; SBP, systolic blood pressure; DBP, diastolic blood pressure; FPG, fasting plasma glucose; TG, triglycerides; TC, total cholesterol; HDL-C, high-density lipoprotein cholesterol; tHcy, total homocysteine; DM, diabetes mellitus; CAD, coronary artery disease; AF, atrial fibrillation. Logistic regression models were adjusted for age, BMI, SBP, DBP, FPG, TG, TC, HDL-C, tHcy, cigarette smoking, alcohol consumption, MTHFR C677T polymorphism, and comorbidity.

### Interactions between sex and other risk factors on stroke risk

Data were reported separately for men and women, and stratified in terms of age-, BMI-, FPG-, blood pressure (BP)-, lipid-, and genotype-specific rates of stroke prevalence. Interactions between sex and other risk factors on stroke risk were statistically significant: age (*P* = 0.005), FPG (*P* = 0.002), SBP (*P* < 0.001), DBP (*P* < 0.001), TG (*P* = 0.004) and tHcy (*P* = 0.038) after adjustment for sex, age, BMI, SBP, DBP, FPG, TC, TG, HDL-C, tHcy, MTHFR C677T polymorphism and history of cigarette smoking, alcohol consumption, DM, hyperlipidemia, CAD, and AF. Subgroup analyses of each risk factor revealed that risk of stroke in those with age ≥55 years, FPG ≥6.1 mmol/L, and TG ≥2.3 mmol/L was higher in women than men (Table 3).

**Table 3:** Stratified analyses of the association between sex and stroke rates in patients with H-type hypertension.

Variables	Women	Men	Women *vs*. Men			*P* for interaction
	no. of events/no. of patients		Coefficient	SE	OR (95%CI)	
Age, years						0.005
<55	104/149	246/309	−0.673	0.331	0.510 (0.266–0.976)	
≥55	369/562	693/1020	0.314	0.153	1.369 (1.014–1.850)	
BMI, kg/m^2^						0.382
<28	396/585	815/1136	0.067	0.152	1.070 (0.795–1.440)	
≥28	77/126	124/193	0.142	0.329	1.153 (0.604–2.198)	
SBP, mmHg						<0.001
<160	190/385	412/754	0.211	0.159	1.235 (0.904–1.687)	
≥160	283/326	527/575	−0.278	0.280	0.757 (0.437–1.310)	
DBP, mmHg						<0.001
<90	147/289	234/463	0.176	0.195	1.193 (0.814–1.748)	
≥90	326/422	705/866	−0.016	0.193	0.985 (0.675–1.436)	
FPG, mmol/L						0.002
<6.1	243/426	559/825	−0.140	0.167	0.869 (0.626–1.207)	
≥6.1	230/285	380/504	0.609	0.247	1.839 (1.133–2.985)	
TG, mmol/L						0.004
<2.3	322/510	720/1013	−0.218	0.156	0.804 (0.592–1.092)	
≥2.3	151/201	219/316	0.479	0.235	1.614 (1.017–2.560)	
TC, mmol/L						0.794
<5.2	242/361	672/936	0.059	0.180	1.060 (0.745–1.509)	
≥5.2	231/350	267/393	−0.034	0.208	0.966 (0.643–1.452)	
tHcy, μmol/L						0.038
≤13.0	172/281	215/318	0.006	0.253	1.006 (0.612–1.652)	
13.1–17.9	183/278	324/483	0.158	0.213	1.172 (0.772–1.777)	
≥18.0	118/152	400/528	0.258	0.294	1.294 (0.728–2.300)	
MTHFR C677T Polymorphism						0.441
CC	136/208	251/374	0.084	0.243	1.087 (0.676–1.750)	
CT	211/313	462/653	0.073	0.208	1.075 (0.715–1.617)	
TT	126/190	226/302	0.177	0.286	1.193 (0.682–2.089)	

Abbreviations: SE, standard error; OR, odds ratio; CI, confidence interval; BMI, body mass index; FPG, fasting plasma glucose; SBP, systolic blood pressure; DBP, diastolic blood pressure; TG, triglycerides; TC, total cholesterol; tHcy, total homocysteine. Logistic regression models were adjusted for sex, age, BMI, SBP, DBP, FPG, TG, TC, high-density lipoprotein cholesterol, tHcy, cigarette smoking, alcohol consumption, MTHFR C677T polymorphism, and comorbidity.

Furthermore, Age (*P* = 0.040), SBP (*P* = 0.001) and DBP (*P* = 0.002) showed significant interactions with tHcy on stroke risk in men. Besides age (P < 0.001), SBP (*P* = 0.003) and DBP (*P* < 0.001), a tHcy-FPG (*P* = 0.019) interaction and a tHcy-TG (*P* = 0.009) interaction could also increase stroke risk in women (Table 4).

**Table 4:** Analysis of tHcy interaction with risk factors in both men and women with H-type hypertension.

Variables	tHcy, μmol/L							*P* for interaction
	≥18.0			13.1–17.9			≤13.0	
	Coefficient	SE	OR (95% CI)	Coefficient	SE	OR (95% CI)		
Men								
Age, years								0.040
<55, n = 309	0.179	0.430	1.196 (0.514–2.781)	−0.029	0.440	0.971 (0.410–2.299)	1.00 (Referent)	
≥55, n = 1020	0.415	0.211	1.514 (1.001–2.292)	−0.094	0.206	0.910 (0.608–1.362)	1.00 (Referent)	
SBP, mmHg								0.001
<160, n = 754	0.283	0.205	1.328 (0.888–1.986)	−0.274	0.205	0.760 (0.509–1.137)	1.00 (Referent)	
≥160, n = 575	0.538	0.427	1.712 (0.741–3.954)	0.343	0.416	1.410 (0.623–3.187)	1.00 (Referent)	
DBP, mmHg								0.002
<90, n = 463	0.334	0.289	1.396 (0.793–2.458)	−0.202	0.280	0.817 (0.472–1.415)	1.00 (Referent)	
≥90, n = 866	0.682	0.256	1.978 (1.197–3.269)	0.079	0.247	1.082 (0.667–1.755)	1.00 (Referent)	
Women								
Age, years								<0.001
<55, n = 149	−0.591	0.616	0.554 (0.166–1.851)	−0.533	0.595	0.587 (0.183–1.884)	1.00 (Referent)	
≥55, n = 562	1.503	0.331	4.496 (2.348–8.606)	0.467	0.233	1.595 (1.010–2.518)	1.00 (Referent)	
SBP, mmHg								0.003
<160, n = 385	0.774	0.331	2.169 (1.133–4.153)	0.158	0.265	1.172 (0.697–1.970)	1.00 (Referent)	
≥160, n = 326	2.056	0.671	7.818 (2.100–29.099)	0.674	0.376	1.962 (0.939–4.098)	1.00 (Referent)	
DBP, mmHg								<0.001
<90, n = 289	0.604	0.398	1.829 (0.838–3.991)	0.420	0.319	1.522 (0.814–2.843)	1.00 (Referent)	
≥90, n = 422	1.446	0.426	4.245 (1.841–9.791)	0.388	0.296	1.474 (0.824–2.635)	1.00 (Referent)	
FPG, mmol/L								0.019
<6.1, n = 426	0.980	0.362	2.663 (1.310–5.413)	0.294	0.265	1.342 (0.799–2.255)	1.00 (Referent)	
≥6.1, n = 285	1.437	0.513	4.206 (1.540–11.486)	0.617	0.394	1.853 (0.857–4.008)	1.00 (Referent)	
TG, mmol/L								0.009
<2.3, n = 510	0.813	0.331	2.254 (1.178–4.312)	0.322	0.253	1.379 (0.840–2.265)	1.00 (Referent)	
≥2.3, n = 201	1.334	0.551	3.795 (1.288–11.182)	0.440	0.410	1.553 (0.695–3.469)	1.00 (Referent)	

Abbreviations: tHcy, total homocysteine; SE, standard error; OR, odds ratio; CI, confidence interval; SBP, systolic blood pressure; DBP, diastolic blood pressure; FPG, fasting plasma glucose; TG, triglycerides. Logistic regression models were adjusted for age, body mass index, SBP, DBP, FPG, TG, total cholesterol, high-density lipoprotein cholesterol, tHcy, cigarette smoking, alcohol consumption, MTHFR C677T polymorphism, and comorbidity.

**Figure 1:**
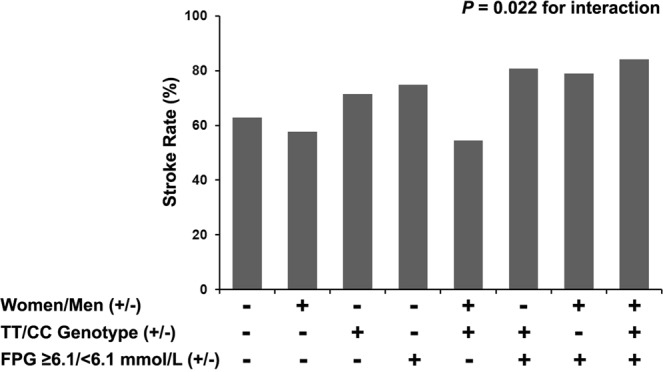
A MTHFR genotype-sex-fasting plasma glucose (FPG) interaction on stroke risk with H-type hypertension.

**Figure 2:**
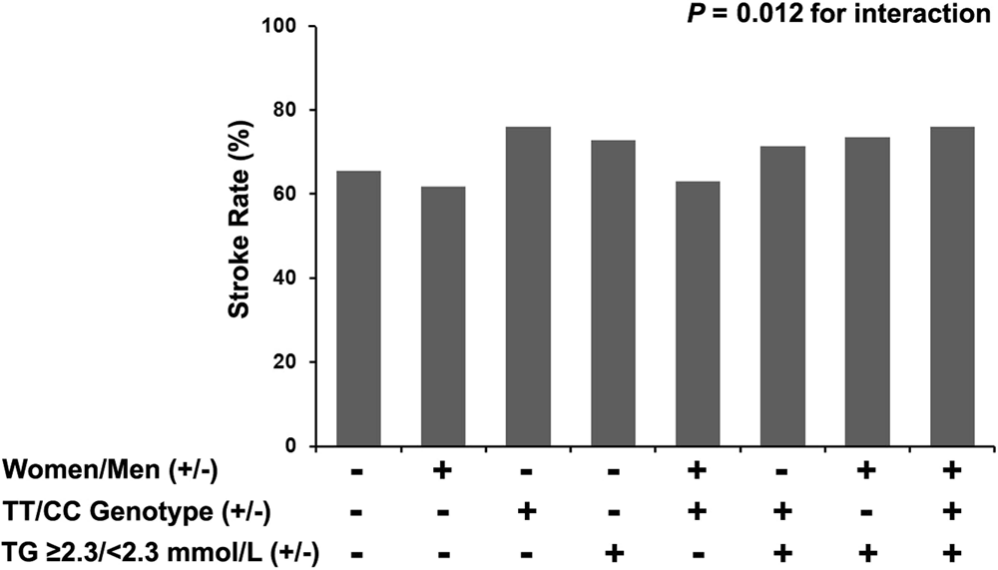
A MTHFR genotype-sex-triglycerides (TG) interaction on stroke risk with H-type hypertension.

**Figure 3:**
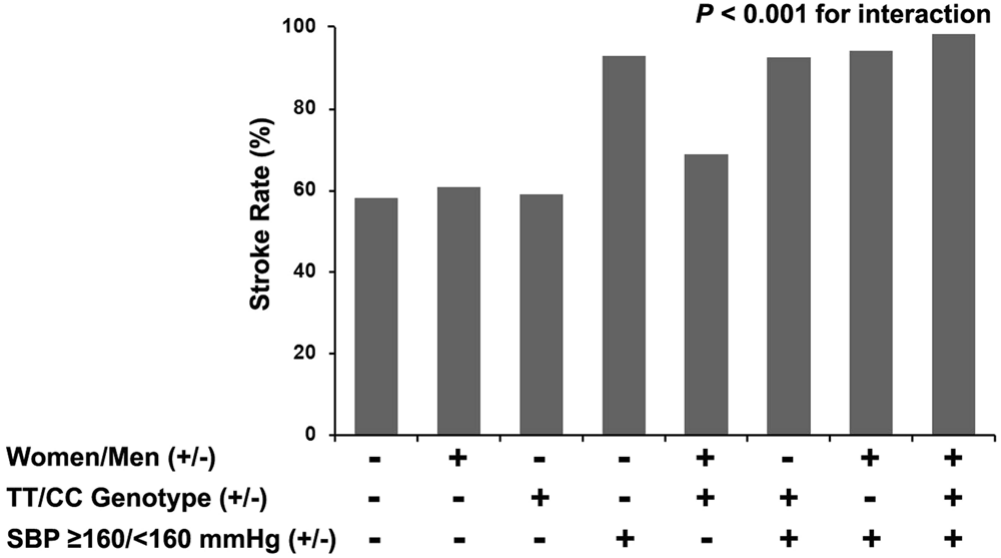
A MTHFR genotype-sex-systolic blood pressure (SBP) interaction on stroke risk with H-type hypertension.

MTHFR genotype-sex-risk factors interactions revealed that stroke risk in women ≥6.1 mmol/L of FPG (Fig. 1) or ≥2.3 mmol/L of TG (Fig. 2) with TT genotype was the strongest, and men interacted with TT genotype and SBP ≥160 mmHg (Fig. 3) on increased risk of stroke (Table 5).

**Table 5:** MTHFR genotype-sex-risk factors interactions on stroke risk in 2040 patients with H-type hypertension.

Variables	Coefficient	SE	OR (95%CI)	*P* for interaction
MTHFR*sex*age				0.288
CT*women*age ≥55 years	0.394	0.250	1.482 (0.908–2.421)	
TT*women*age ≥55 years	0.199	0.301	1.221 (0.676–2.203)	
MTHFR*sex*BMI				0.692
CT*women*BMI ≥28 kg/m^2^	0.236	0.381	1.266 (0.601–2.670)	
TT*women*BMI ≥28 kg/m^2^	0.305	0.463	1.356 (0.548–3.358)	
MTHFR*sex*FPG				0.022
CT*women*FPG ≥6.1 mmol/L	0.588	0.307	1.800 (0.987–3.285)	
TT*women*FPG ≥6.1 mmol/L	0.879	0.400	2.410 (1.100–5.276)	
MTHFR*sex*DBP				0.946
CT*women*DBP ≥90 mmHg	−0.081	0.263	0.922 (0.551–1.545)	
TT*women*DBP ≥90 mmHg	−0.063	0.321	0.939 (0.501–1.763)	
MTHFR*sex*TG				0.012
CT*women*TG ≥2.3 mmol/L	0.807	0.348	2.240 (1.133–4.428)	
TT*women*TG ≥2.3 mmol/L	0.918	0.429	2.505 (1.081–5.806)	
MTHFR*sex*TC				0.786
CT*women*TC ≥5.2 mmol/L	0.112	0.263	1.119 (0.669–1.872)	
TT*women*TC ≥5.2 mmol/L	−0.141	0.321	0.869 (0.463–1.631)	
MTHFR*sex*tHcy				0.453
CT*women*tHcy (13.1–17.9) μmol/L	0.288	0.276	1.333 (0.776–2.290)	
TT*women*tHcy (13.1–17.9) μmol/L	−0.241	0.357	0.786 (0.390–1.582)	
CT*women*tHcy ≥18.0 μmol/L	−0.204	0.428	0.815 (0.353–1.885)	
TT*women*tHcy ≥18.0 μmol/L	0.427	0.392	1.532 (0.710–3.306)	
MTHFR*sex*SBP				<0.001
CT*men*SBP ≥160 mmHg	1.050	0.281	2.857 (1.648–4.951)	
TT*men*SBP ≥160 mmHg	1.027	0.423	2.792 (1.220–6.391)	

Abbreviations: SE, standard error; OR, odds ratio; CI, confidence interval; BMI, body mass index; FPG, fasting plasma glucose; DBP, diastolic blood pressure; TG, triglycerides; TC, total cholesterol; tHcy, total homocysteine; SBP, systolic blood pressure. Logistic regression models were adjusted for sex, age, BMI, SBP, DBP, FPG, TG, TC, high-density lipoprotein cholesterol, tHcy, cigarette smoking, alcohol consumption, MTHFR C677T polymorphism, and comorbidity.

## Discussion

The present study has shown that most independent risk factors for stroke in women were similar to those in men among patients with H-type hypertension, including DM, AF, smoking, increased level of SBP and tHcy, as well as decreased HDL-C. DBP-specific risk of stroke was unique to men. Interactions between sex and other risk factors on stroke risk were statistically significant: age, FPG, SBP, DBP, TG and tHcy. Furthermore, an interaction between tHcy and other factors also modulated the risk of stroke, including age, SBP and DBP in men, and age, SBP, DBP, FPG, and TG in women. At the same time, although TT genotype did not have an independent effect on stroke, it could interact with sex and FPG, TG and SBP to increase stroke.

H-type hypertension has a large negative impact on society. Besides an independent effect, a multiplicative effect of hypertension and HHcy substantially increases the risk for stroke^18^. Sex differences in incident and recurrent stroke, temporal patterns of stroke, and outcomes after stroke have been reported in published studies^19^. As noted previously, females have a lower ischemic stroke incidence over much of their life span than their age-matched male counterparts, which was changed in those aged >85 years^20^. Stroke risk factors include reproductive factors that are unique to women and obesity, metabolic syndrome, and AF that are more common in women than men^21^. Data on the epidemiology of stroke in South, East, and South-East Asia indicated that a high prevalence of hypertension, DM and tobacco smoking was seen among men, whereas a high prevalence of hypercholesterolemia, inactivity and obesity was seen among women^22^.

Our study found effects of DM, AF, smoking, increased level of SBP and tHcy, as well as decreased HDL-C on stroke risk were independent, after multivariate adjustment for risk factors. However, DBP-specific risk of stroke was independent in men but not in women. As the independent risk factors for stroke were similar in men and women, an in-depth analysis should be considered to investigate the interaction between sex and other stroke risk factors in patients with H-type hypertension. As a result, interactions between sex and other risk factors included age, FPG, SBP, DBP and TG revealed that the association of higher SBP and DBP with stroke risk was the strongest in men. In addition, a higher risk of stroke in women was associated with higher age, FPG, TG and tHcy compared with men. Several studies have indicated that BP in women is lower across much of their lifespan than their age-matched men. Higher prevalence of hypertension is observed in men than women before 45 years, but the prevalence of hypertension becomes higher in postmenopausal women than men after 55 years^23^. Among participants with hypertension after 80 years in the Framingham Heart Study, 38% of men but only 23% of women showed a BP of <140/90 mm Hg^24^. This result indicated that BP control rates that were poorer in high-risk elderly women than men, and might provide greater insight into the effects of hormonal status on vascular function and BP in stroke prevention, especially among older, postmenopausal women^25^. Sex differences in BP control rates could partly explain our results that the prevalence of stroke was higher in men than women before 53 years, but an age-related increase in stroke rates showed no differences between men and women.

Homocysteine is a well-established marker of increased stroke risk. Moreover, there is increasing evidence that elevation of Hcy is associated with an increased risk for arteriosclerosis^26^, myocardial infarction, venous thrombosis, and neural tube defects. In a recent meta-analysis of 9 prospective studies, elevated Hcy levels were associated with an increased risk for ischemic strokes and recurrent strokes, however, these had no distinct association with hemorrhagic strokes^27^. Data from the China Stroke Primary Prevention Trial showed that elevated tHcy was associated with death from any cause and the association was subject to effect modification by MTHFR genotypes among 20,424 hypertensive patients^28^. Data of a retrospective investigation indicated that increased Hcy was independently associated with 85% increased risk of death about five years after ischemic stroke in women but not in men^29^. In the China Antihypertensive Trial in Acute Ischemic Stroke (CATIS) study, elevated tHcy was positively associated with increased risks of death and major disability 3 months after acute ischemic stroke in women but not in men^30^. Many clinical trials confirmed that a reduction in total homocysteine was associated with a decrease in the risk of stroke. However, reduction in homocysteine has not been shown to prevent recurrent stroke in adults with a recent ischemic stroke or TIA who are known to have mild to moderate hyperhomocysteinemia^31^. Our study demonstrated that tHcy showed an independent effect, and also interactions with other factors to modulate the risk of stroke. These risk factors included age, SBP and DBP in men, and age, SBP, DBP, FPG, and TG in women.

MTHFR C677T mutation is associated with many diseases, such as stroke^32^, CAD^33^, and thrombosis^34^. Homozygous form (TT) of the MTHFR C677T is prevalent in 10% of the population. This genotype has also been shown to correlate with elevated Hcy levels^35^. Our study observed that TT genotype rates were higher both in men and women than the above investigation demonstrated, and stroke rates of TT subgroup were higher in men than women among patients with H-type hypertension. However, there was no association between TT genotype and stroke risk after adjustment for multiple risk factors both in men and women. We assessed the complexities of the interactions of risk factors with a stratified analysis. Differences in interactions between TT genotype and risk factors in stroke risk among men and women are recognized. The association of higher SBP with stroke risk was the strongest in male patients with TT genotype, and certain interactions between TT genotype and FPG, or TT genotype and TG are specific to women’s health. Both sex and genotype are non-modifiable risk factors for stroke. Therefore, successful reduction of SBP, FPG and TG may be much more important to decrease stroke risk.

### Study Limitations

This retrospective study had several limitations. First, although logistic regression model was performed as a means of controlling for differences in characteristics of the patients and their diseases, we had no medication data on admission, such as antihypertensive, lipid-lowering, hypoglycemic and even anti-platelet agents. That might have had an influence on distinguishing the relationship between sex and stroke risk. Second, due to nature of a cross-sectional study, the time of stroke onset was unknown in our study. Data were analyzed retrospectively after onset of stroke. Precise information about the pre-stroke status was unavailable. Both of these factors limit conclusions of a causal relationship between risk factors and outcome, which will be illustrated by the ongoing follow-up phase of our study. Third, because this was a hospital-based study based on data from a single medical center, characteristics of the present subjects might differ from those of the general community. Thus, a prospective multicenter registry trial with a large sample population is needed to clarify our results.

## Conclusions

Risk stratification in H-type hypertension that account for sex-specific differences is useful to stratify risk of stroke and assist clinicians in the decision to select a reasonable therapeutic option for high-risk patients. Our study shows that, although most independent risk factors for stroke in women are similar to those in men, sex differences in interactions between individual factors that need to be considered in predicting overall risk are much more important. For example, tHcy, but not MTHFR C677T, is an independent risk factor for stroke. However, MTHFR C677T can interact with sex and other factors to increase stroke risk.
